# Executive Functions in Students With Depression, Anxiety, and Stress Symptoms

**DOI:** 10.18869/nirp.bcn.8.3.223

**Published:** 2017

**Authors:** Bita Ajilchi, Vahid Nejati

**Affiliations:** 1. Department of Psychology, Faculty of Humanities and Social Sciences, Science and Research Branch, Islamic Azad University, Tehran, Iran.; 2. Department of Psychology, Faculty of Education and Psychology, Shahid Beheshti University, Tehran, Iran.

**Keywords:** Executive functions, Selective attention, Shifting attention, Sustained attention, Depression

## Abstract

**Introduction:**

This study aimed to investigate and compare the executive functions of students with depression, anxiety, and stress symptoms with those functions in healthy ones.

**Methods:**

This study was a comparative and non-clinical analysis. The study population comprised all students of Shahid Beheshti University, Tehran, Iran. A total of 448 students were recruited using convenience sampling method. They were also screened using the Depression Anxiety Stress Scales (DASS) test comprising 21 items. Of study participants, 30 people were depressed, 27 had anxiety, and 15 suffered from stress. Then, 50 control people were matched with them. Next, both groups were compared using the Stroop test, Wisconsin card sorting, and cognitive ability test.

**Results:**

Using MANOVA test, data analysis revealed no significant differences among 4 groups with regard to selective attention and shifting attention. Depressed group reacted rapidly as opposed to the anxiety group with regard to measures of shifting attention and cognitive abilities; it was observed that the memory, inhibition control, planning, and flexibility of the healthy group were better than those of the 3 other groups.

**Conclusion:**

The findings of this research raised specific issues in relation to the role of depression, anxiety, and stress in the disruption of the executive functions of sufferers. Selective and shifting attention and cognitive abilities are specifically affected in this regard. Meanwhile, the role of stress in impairing decision making and the major role of anxiety in impairing sustained attention was shown to be considerable.

## 1. Introduction

Today, a wealth of theories exist with regard to definitions of cognitive processes and suitable methods to measure executive functions (Wood, 2013). Welsh and Pennington (1988) defined executive functions as a solution to obtain future targets. They specified 4 key elements of intention/target orientation, inhibition, planning, and working memory for these executive functions. Miyake, Friedman, Emerson Witzki, Howerter, and Wager (2000) introduced various theories concerning executive functions, including 3 dimensions of attention shifting, updating the working memory, and response inhibition. In general, executive functions can be seen as cognitive processes such as the ability to sustain and shift attention, dominant response inhibition, and maintenance of information in the working memory, as well as planned responses (Pennington & Ozonoff, 1996).

Many studies suggest that depression is associated with impairment of executive functions (Alves et al., 2014; Otte et al., 2015) and people with depression function more weakly compared to healthy people in executive function tests (Doumas, Smolders, Branfaut, Boukaert and Krampe, 2012). It seems that marked dysfunction in frontal regions of depressed people is associated with their impaired executive functions (Alves et al., 2014).

Review of the research literature concerning the study of the relationship between depression and executive dysfunction brings about controversial results. Some studies have found that depression is associated with deficiencies in executive functions (e.g., Brooks, Iverson, Sherman, & Roberge, 2010; Dulay, Busch, Chapin, Jehi, & Najm, 2013; Vergara-Lopez, Lopez-Vergara, & Colder, 2013; Wagner, Alooy, & Abramson, 2014). However, other studies did not observe these deficiencies and impairments in depressed people (Watkins, & Brown, 2002; Smitheman, Huerkamp, Miller, Houle, & O’Jile, 2007; Holler, Kavanaugh, & Cook, 2013; Fujii et al., 2013). Based on such controversy, it seems that seriousness of executive dysfunctions depends upon the severity of depression (Bredemier, 2012; Holler et al., 2013). In spite of a strong relationship between depression and anxiety, little attention has been given to the relationship between executive functions and anxiety.

It is generally stated that the studies carried out in this area have no specific experimental framework utilized in evaluating executive functions in cases of anxiety. In later studies concerning these kinds of cases, mixed results were obtained (Vadnais, et al., 2013). For example, Billingsley-Marshall et al. (2013) and Visu-Petra, Miclea and Visu-Petra (2013) found that people with high levels of anxiety had deficits in their executive functions. However, Smitheman et al. (2007) and Fuji et al. (2013) could not find a relationship between self-reported anxiety symptoms and impairment of executive functions. Apparently, the degree of executive function deficit is related to the intensity of anxiety symptoms (Bredemeier, 2012). Executive functions are possibly susceptible to impairment by stress (Blair et al., 2011).

It is suggested that the relative capacity of executive functions can predict the presence of anxiety. Previous studies have not yet clarified the relationship between the executive functions and stress (Hendrawan, Yamakawa, Kimura, Murakami, & Ohira, 2012). In this respect, some studies found that high levels of stress are associated with low levels of executive functions (Blair et al., 2011; Hendrawan et al., 2012). However, Wudarczyk (2010) found that when people are faced with stress before a task, their executive functions are not affected. Due to the inconsistencies in previous research, further studies on executive functions and depression, anxiety, and stress are necessary. In addition, no studies have been found so far comparing these 3 disorders together as well as comparing these groups with healthy people and in non-clinical subjects. Accordingly, the research question is raised as how executive functions are carried out in students with depression, anxiety, and stress symptoms.

## 2. Methods

### 2.1. Participants

This is a comparative cross-sectional and non-clinical study. The study population comprised all students of Shahid Beheshti University, Tehran, Iran, of them 448 students (both female and male) were selected. The subjects were selected using convenience sampling method. Amongst the research population (448 students), tests of Depression Anxiety Stress Scales (DASS) were administered. With its 21 questions, 30 students with depression, 27 with anxiety, and 15 with stress were uncovered (cutoff point for depression test: 14, anxiety test: 10, and stress test: 19). The rest were healthy students that among them 50 students matched in terms of gender, age, marital status and occupations were selected as the control group.

The mean age of the sample group was 22.61 years (standard deviation 2.5). It should be noted that 30 individuals were males and 92 females. All groups were tested using Stroop Test, Wisconsin card sorting test, and questionnaires concerning comparison of cognitive abilities. The most important moral principles taken into consideration in the present study were as follows: 1) Getting consent from the participants before doing the research. All students had a right to privacy when participating in research. Also we were sure about the participants’ rights during the research. 2) This study was approved by Research Ethics Board (REB) of Shahid Beheshti University for the ethical and regulatory compliance of research involving human subjects. We were committed to protect the rights and welfare of subjects enrolled in research activities.

### 2.2. Instruments

#### 2.2.1. Demographic questionnaire

A researcher-made questionnaire was used to determine the subjects’ demographics, including age, gender, education, and marital status.

#### 2.2.2. Depression, Anxiety and Stress Scale

This scale was designed by Lavibond and Lavibond in 1995; it is a collection of 3 self-report scales for measuring the intensity of depression, anxiety and stress symptoms within one week. Every subscale encompasses 7 items being scored from 0 (this is not true about me) to 3 (this is true about me). Since, this form of the questionnaire (21 questions) is a short form of the large scale (42 questions), the final score of every subscale should be two-folded and then be defined by the use of categorization score table to determine the severity of symptoms (Lavibond & Lavibon 1995).

Antony, Bieling, Cox, Enns and Swinson (1998) produced a factorial analysis of the scale and found out that about 98% of the variance of the whole scale was explained by these 3 factors. The values of the stress, depression and anxiety factors of this research are 9.07, 2.89 and 1.23, respectively; and α coefficients are 0.97, 0.92, and 0.95 for these factors, respectively. Also, the correlation coefficient between depression and stress is 0.48, anxiety and stress as 0.53, and anxiety and depression as 0.28. In Samani and Jokar (2007) research, the reliability values of the test-retest for depression, anxiety and stress are 0.80, 0.76, and 0.77, respectively and the Cronbach α values are 0.81, 0.74, and 0.78, respectively.

#### 2.2.3. Stroop Test

This test is one of the most applicable tests for the measurement of selective attention and response inhibition (Chan, Chen, & Law, 2006; Bozikas, Kosmidis, Kiosseoglou, & Karavatos, 2006). In this study, a computer-based version was run which included three stages. The first step tests cohesive struggles. The names of 4 colors written in black appear in the center of the monitor and the participant has to rapidly press one of the keys representing the colors. In the second stage, the names of 4 colors appear in the center of the monitor written in their own colors. The participant should then press a key on the keyboard denoting this color. The third step is related to intervention struggle; the names of four colors appear on the monitor in a different color to the one they spell.

The participant has to press the color of the font instead of the word. The indices measured in this test are accuracy (number of correct responses) and speed (mean time of correct responses reaction in versus of stimulant per thousand seconds). The reliability of the Stroop test was reported based on Otello and Graf (1995) (Cited in Karimi Ali Abadi, Kafi and Farahi 2010), in a test-retest method for all three task as follows, 0.01, 0.83, and 0.90, respectively. Ghadiri, Jazayeri, Ashayeri and Ghazi Tabatabaiee (2006) reported the reliability of test-retest of the three tasks as being, 0.6, 0.83, and 0.97. Penner, Kobel, Stöcklin, Weber, Opwis and Calabrese (2012) compared the performance in the original task with computerized version in children and adult. All two versions showed high test-retest reliability and are able to elicit interference effects.

#### 2.2.4. Wisconsin Card Sorting Test (WCST)

This test is a very common test to evaluate executive functions (Rossi, Arduini, Danelluzzo, Bustini, Prosperini, & Stratta, 2000). It is also applied in examining the executive functions of brain such as attention shifting (Sergeant, Geurts, & Oosterlaan, 2002), flexibility (Tabares-Seisdedos, Balanza-Martinez, Salazar-Fraile, Selva-Vera, Leal-Sercos, & Gomez-Beneyto, 2003), problem-solving (Silverstein, Lefteros, & Turnball, 2003) and the concept of the formation and ability to overcome or persevere (Chan et al., 2006). In this test, the participant should keep a concept or principle received in the test step at frequent cycles and when the principles of sorting change, the early concepts would change in this regard (Ghasemzadeh, KaramGhadiri, Sharifi, Norouzian, Mojtabaei, & Raminea Ebrahimkhani, 2005).

In this study, a computerized version of the Wisconsin test was utilized. This test has 64 different cards, on which there are diagrams of a triangle, star, cross or a circle as well as a number from 1 to 4. Moreover, these cards are colored blue, red, yellow, and green. Hence, the cards have a figure (one of four types), a number (from 1–4), and a color (blue, red, yellow, or green). The combination of these gives 64 variations. In other words, every card represents a unique design which is not repeated in any other card. The scores of the test are as follows: 1-number of the correct response, 2- score of perseveration error (this error occurs when the respondent continues sorting based on former principle or a wrong guess, or despite receiving feedback from the assessor trying to avert the incorrect response), and 3- number of clusters, which refers to correct sorting based on three main colors, figure and number ranging from 0 to 3 fluctuating in this regard. The validity of this test is above 0.86 for measuring cognitive deficiencies after traumatic brain events (Lezak, 2004) and its reliability in the research carried out by Spreen and Strauss (1991) (Cited in Karimi Ali Abad et al., 2010) was reported as 0.83 based on the agreement coefficient of assessors. Naderi (1994) (Cited in Karimi Ali Abad et al., 2010) reported the reliability of the test using test-retest method as 0.85.

#### 2.2.5. Cognitive Abilities Test

This test was designed by Nejati (2013); this is a questionnaire with 30 items saturated by 7 factors. Each factor has at least 3 options based on the Likert-type scale of 5 multiple-choices ranging from 1 (never) to 5 (always). The reliability of the questionnaire was 0.834 using Cronbach α coefficient test. In studying the reliability of the questionnaire via a test-retest method, the Pearson correlation coefficient was obtained as 0.865 at 0.0001 level of significance.

### 2.3. Statistical analysis

In order to compare the functions of selective attention, Stroop test was applied. To investigate the function of shifting attention, WCST was utilized. Moreover, the related test was run to measure the cognitive abilities of the participants. In addition to descriptive indicators of the study variables, to analyze the data obtained and compare the scores of the four groups in each instrument, multivariate ANOVA and Tukey post hoc test were used. We performed a power analysis for MANOVA test (4 groups). The results of power analysis showed that the number of participants was sufficient (power estimated more than 0.8).

## 3. Results

Demographic characteristics of the participants are given in Table 1. Stroop test was used to compare the selective attention functions and the scores of four groups were compared using MANOVA in this case. The results are given in Table 2. As shown in Table 2, the four groups do not have significant differences in terms of their mean score of correct responses (accuracy) and their reaction time (speed) in any step of the Stroop test (P<0.05). The shifting of attention functions were studied using Wisconsin test. In order to evaluate the scores of the four groups, the results of MANOVA are given in Table 3. According to Table 3, there are no significant differences among all groups. According to Table 4, there is a significant difference in all cognitive elements except for social cognition (P<0.05). Tukey test was used to study the differences among the groups; the results are presented in Table 5.

**Table 1.** Demographic characteristics.

	**Range**	**No.**	**%**
Age, y	18–22	74	60.7
	23–27	44	36.1
	28–32	2	1.6
	33–38	2	1.6
Sex	Male	30	24.6
	Female	92	75.4
Marital status	Single	85	69.7
	Married	37	30.3

**Table 2.** MANOVA to compare the selective attention functions (Stroop Test) among the groups.

**Stroop Test**	**Depressed Group n=30**	**Anxiety Group n=27**	**Stress Group n=15**	**Healthy Group n=50**	**MANOVA**	

	**Mean±SD**	**Mean±SD**	**Mean±SD**	**Mean±SD**	**F**	**P**
Correct response to step 1	99.47±1.383	99.11±2.025	100.00±0.000	99.12±2.715	0.808	0.492
Correct response to step 2	98.27±4.160	98.81±1.861	99.47±1.407	99.20±1.807	1.085	0.358
Correct response to step 3	94.40±16.429	97.63±5.350	92.27±25.633	95.52±13.392	0.461	0.710
Reaction time for step 1	1.13±0.434	1.15±0.362	1.00±0.000	1.10±0.303	0.708	0.549
Reaction time for step 2	1.07±0.254	1.11±0.320	1.00±0.000	1.00±0.000	2.271	0.084
Reaction time for step 3	1.30±0.466	1.19±0.396	1.00±0.378	1.24±0.847	0.799	0.497

**Table 3.** MANOVA to compare the shifting attention (Wisconsin Test) in groups.

**Wisconsin Test**	**Depressed Group n=30**	**Anxiety Group n=27**	**Stress Group n=15**	**Healthy Group n=50**	**ANOVA**	

	**Mean±SD**	**Mean±SD**	**Mean±SD**	**Mean±SD**	**F**	**P**
Clusters	2.97±0.928	2.96±1.055	3.27±1.100	3.06±1.038	0.354	0.787
Correct response	33.80±8.323	9.116±10.254	37.67±1.407	34.44±9.422	0.706	0.550
Perseverance error	19.97±6.100	20.70±8.615	17.87±8.951	18.04±6.937	1.036	0.379

**Table 4.** MANOVA to compare the cognitive abilities test among groups.

**Cognitive Abilities Test**	**Depressed Group n=30**	**Anxiety Group n=27**	**Stress Group n=15**	**Healthy Group n=50**	**ANOVA**	

	**Mean±SD**	**Mean±SD**	**Mean±SD**	**Mean±SD**	**F**	**P**
Memory	23.700±3.495	22.031±4.229	22.933±4.527	25.860±2.770	7.827	0.0005
Inhibitory control	18.667±4.063	19.630±3.510	17.467±4.138	22.880±3.701	12.475	0.0005
Decision making	17.500±3.422	17.148±3.780	14.333±3.016	19.200±2.969	9.037	0.0005
Planning	9.400±2.990	9.630±3.399	9.133±3.204	11.780±2.674	6.170	0.001
Sustained attention	8.633±2.399	8.370±2.559	8.133±2.560	9.820±2.007	3.744	0.013
Social cognition	11.633±1.426	11.444±1.968	11.667±1.759	11.200±1.457	0.604	0.614
Flexibility	13.367±3.296	13.038±3.044	12.200±3.299	15.820±2.577	9.449	0.0005

**Table 5.** Tukey Test to study differences between groups.

**Tukey Test**	**Group 1**	**Group 2**	**Means Difference**	**SE**	**P**
Memory	Healthy	Depressed	2.160	0.818	0.046
		Anxiety	3.823	0.464	0.0005
		Stress	2.927	1.043	0.030
	Depressed	Anxiety	1.662	0.940	0.294
		Stress	0.767	1.121	0.903
	Anxious	Stress	−0.896	1.141	0.861
Inhibitory control	Healthy	Depressed	4.213	0.879	0.0005
		Anxiety	3.250	0.909	0.003
		Stress	5.413	1.121	0.0005
	Depressed	Anxiety	−0.963	1.009	0.776
		Stress	1.200	1.203	0.751
	Anxious	Stress	2.162	1.225	0.296
Decision making	Healthy	Depressed	1.700	1.757	0.118
		Anxiety	2.051	0.783	0.048
		Stress	4.867	0.966	0.0005
	Depressed	Anxiety	0.352	0.870	0.987
		Stress	3.167	1.038	0.015
	Anxious	Stress	2.815	1.057	0.043
Planning	Healthy	Depressed	2.380	0.690	0.004
		Anxiety	2.150	0.714	0.016
		Stress	2.645	0.880	0.017
	Depressed	Anxiety	−0.2230	0.793	0.991
		Stress	0.267	0.945	0.992
	Anxious	Stress	0.496	0.962	0.955
Sustain attention	Healthy	Depressed	1.187	0.532	0.121
		Anxiety	1.450	0.550	0.046
		Stress	1.678	0.678	0.067
	Depressed	Anxiety	0.263	0.611	0.937
		Stress	0.500	0.729	0.902
	Anxious	Stress	0.237	0.742	0.989
Flexibility	Healthy	Depressed	2.453	0.684	0.003
		Anxiety	2.783	0.707	0.001
		Stress	3.620	0.871	0.0005
	Depressed	Anxiety	0.330	0.785	0.975
		Stress	1.167	0.936	0.599
	Anxious	Stress	0.837	0.953	0.816

As shown in Table 5, there are significant differences between elements of the memory, inhibition control, planning and flexibility of three groups with the healthy group. The memory, inhibition control, planning and flexibility of the healthy group are better in comparison to other groups. Moreover, no difference was found between the various elements within the 3 disease groups. With regard to the decision making element, there is a significant difference among the healthy group and the anxiety and stress groups. There is also a difference among the depressed and the anxiety groups in comparison to the stress group. The decision-making of the healthy group is better than the anxiety and the stress groups. Likewise, the decision making of the depressed and the anxiety groups is better than the stress group. Regarding the tests of sustained attention, there is a significant difference among the healthy group and the anxiety sufferers, with healthy people having a better sustained attention.

## 4. Discussion

The results of this study substantiated that no significant differences exist among the four groups with regard to selective attention and shifting attention. After studying cognitive abilities, it became clear that the memory, inhibition control, planning and flexibility of the healthy group were better than all other groups. Also, the decision making of the healthy group was better than individuals who suffered from stress and anxiety. The decision-making of the depressed and anxiety groups was better than the stress group as well. In addition, the sustained attention of healthy people was only better than that of anxiety individuals.

The results showed that there were no differences between students of the study groups with regard to selective attention. This finding is consistent with that of the study conducted by Mogg, Bradly, Bono, and Painter (1997). They postulated that attentional bias for threat does not appear in non-clinical anxiety. However, it is inconsistent with Ellenbogen, Schwartzman, Stewart and Walker (2002) with regard to impairment of selective attention under stress status and with the study by Wells and Beevers (2010) about deficits of selective attention in individuals with depressive symptoms.

The finding of this study about differences among groups in shifting attention is not in line with the results of Bredemeier (2012) indicating that the shifting attention of depressed people is worse than people with anxiety. Also inconsistent with Vergara-Lopez et al. (2013), which showed that inhibition of attention shifting was related to depression and anxiety. However, in the studies of Watkins and Brown (2002), Smitheman et al. (2007), Holler et al. (2013), and Fouji et al. (2013) such impairments were not observed in depressed people.

Also, there are not differences among depressed and healthy groups with regard to the sustained attention. This finding is inconsistent with the study carried out by Wagner et al. (2014). They predicted that sustained attention in depressed individuals was worse than that in healthy ones.

Also, this study showed that the depressed group was worse than healthy people in terms of the measures of memory, inhibition control, planning, and flexibility. This conclusion is concordant with the research of Brooks et al., (2010) as well as Dulay et al. (2013) but it does not agree with the findings of Smitman et al. (2007), Fuji et al. (2013), and Watkins and Brown (2002). Moreover, both groups suffering from anxiety and stress have worse measures of decision making affairs in comparison with the healthy group. This finding is in agreement with the results of Bilingsli-Marshal et al. (2013) and Visu-Petra et al. (2013); however, it is not in agreement with the research by Smitheman et al. (2007), Fuji et al. (2013) as well as Watkins and Brown (2002). In addition, anxiety and stress groups are worse than the healthy group both in the above components and in decision making. This finding concerning anxious people is consistent with the research by Billingsley-Marshall et al. (2013), but is in disagreement with the research by Smitheman et al. (2007) and Fuji et al. (2013). Concerning people with stress, this study result is consistent with that of Blair et al. (2011) and Hendrawan et al. (2012), but is not in agreement with Wudarczyk (2010).

Lack of a difference among the depressed, anxious and stressed people compared to healthy people, with regard to the other executive elements may be related to the intensity of symptoms. These elements include the selective and sustained attention (except for anxiety group in sustained attention). A wealth of studies that observed a difference among one of these three disease groups and healthy people carried out tests on individuals with up to moderate symptoms while in this study the symptoms of the population was below moderate at three groups. In this research, the process of decision-making in the three groups is worse than that in the healthy individuals. This result is consistent with the study of Anderson, Arnold, Angus and Bryce (2009) which assessed the impact of depression and anxiety on the process of decision making and the research carried out by Stracke, Polzer, Wolf and Brand (2011) which studied stress.

In addition, amongst the three groups with related symptoms, the decision making of stressed people was worse than depressed and anxiety groups. This was probably due to the disruption of decision making in people with depression and anxiety, the only dysfunction happens in emotions (Paulus and Yu, 2012) but the stress and decision making are complexly related to each other not only at behavioral level but also at neural level since the brain areas related to the decision making are susceptible to changes-induced stress (Stracke & Brand, 2012).

The final point in the present study for discussion was the fact that people with anxiety sustained their attention worse than healthy people. This finding is consistent with the research conducted by Ballard (1996) based on the negative impact of anxiety on sustained attention. However, it is not consistent with the results obtained by Arjmandi Beghlar, Zarenezhad Ashkzari, Nejati, Shah Mansouri, and Raoufi Ahmad (2013). This might be due to the differences of the sample population because they carried out their research on cardiac patients within the clinical environment whilst the present study was carried out on students. In general, sustained attention is related to the maintenance of vigilance over time (Bishop, Lau, Shapiro, Carlson, Anderson, & Carmody, 2003) and is considered a basic requirement for processing information. Almost, all aspects of cognitive-processing such as encoding, storage, planning and problem solving happen within the periods of sustained attention (Richards & Hunter, 1998; Cited in Ung et al., 2010). It should be noted that people with deficiencies in sustained attention may not be able to adapt to the demands of the environment, and may not perform well in this setting by inhibiting inappropriate behaviors (DeGangi and Proges 1990; Cited in Ung et al., 2010). It is obvious that in maintaining vigilance to threatening stimuli, people with anxiety can lower their sustained attention making operation of other aspects of their executive functions difficult as well as lowering their ability to adapt to their environmental demands in this process.

The findings of this research raised specific issues in relation to the role of depression, anxiety, and stress in the disruption of executive functions of sufferers. Selective and shifting attention and cognitive abilities are specifically affected in this regard. Meanwhile, the role of stress in decision making impairment and the major role of anxiety in deficit of sustained attention were shown to be considerable in this regard. For this reason, all students were recommended for suitable treatment. In addition, none of the groups had difficulties in comparison to healthy people in the cognitive neurological tests; however, they showed dysfunction in the questionnaire test. In other words, there was no problem with respect to the executive functions of these people but they may have had these problems from their own viewpoints. Much of these functional deficiencies may arise from a lack of confidence or the perfectionism of such people; for that reason, these issues should comprehensively be examined in future studies.

Since all differences in the prepared research have been gained through cognitive abilities questionnaire and no difference was observed in objective tests, it seems that differences are the result of questionnaire self-reporting bias. In fact, the participants have the mentioned defects in executive functions regarding to their idea while objective tests did not confirm the mentioned differences in any groups. Non-clinical symptoms should also be taken into account as disorders in executive functions of depressed and restless individuals gained through clinical samples in most of the previous studies. A dearth of research on non-clinical samples is clearly seen in this field. Accordingly, related subjects are recommended for future studies.
